# Plasma Lipoprotein(a) Levels Are Associated with Mild Renal Impairment in Type 2 Diabetics Independent of Albuminuria

**DOI:** 10.1371/journal.pone.0114397

**Published:** 2014-12-09

**Authors:** Jennie Lin, Muredach P. Reilly, Karen Terembula, F. Perry Wilson

**Affiliations:** 1 Renal Electrolyte and Hypertension Division, Department of Medicine, Perelman School of Medicine, University of Pennsylvania, Philadelphia, PA, United States of America; 2 Cardiovascular Institute, Perelman School of Medicine, University of Pennsylvania, Philadelphia, PA, United States of America; 3 Section of Nephrology, Program of Applied Translational Research Department of Medicine, Yale School of Medicine, Yale University, New Haven, CT, United States of America; University of Louisville, United States of America

## Abstract

**Background:**

CKD, an independent risk factor for CV disease, increases mortality in T2DM. Treating modifiable CV risk factors decreases mortality in diabetics with microalbuminuria, but the role of early CV prevention in diabetics with mild CKD by GFR criteria alone remains unclear. The purpose of this study was to probe whether T2DM patients with mild GFR impairment have atherogenic lipid profiles compared to diabetic counterparts with normal renal function.

**Methods:**

In the Penn Diabetes Heart Study (PDHS), a single-center observational cohort of T2DM patients without clinical CVD, cross-sectional analyses were performed for directly measured lipid fractions in 1852 subjects with eGFR>60 mL/min/1.73 m^2^ determined by the CKD-EPI equation (n = 1852). Unadjusted and multivariable analyses of eGFR association with log-transformed lipid parameters in incremental linear and logistic regression models (with eGFR 90 mL/min/1.73 m^2^ as a cut-point) were performed.

**Results:**

Mild GFR impairment (eGFR 60–90 mL/min/1.73 m^2^, median urinary ACR 5.25 mg/g) was associated with higher log-transformed Lp(a) values (OR 1.17, p = 0.005) and with clinically atherogenic Lp(a) levels above 30 mg/dL (OR 1.35, p = 0.013) even after full adjustment for demographics, medications, metabolic parameters, and albuminuria. Logistic regression demonstrated a trend towards significance between worse kidney function and apoB (p = 0.17) as well as apoC-III (p = 0.067) in the fully adjusted model.

**Conclusions:**

Elevated Lp(a) levels have a robust association with mild GFR impairment in type 2 diabetics independent of race, insulin resistance, and albuminuria.

## Introduction

Affecting over 20 million patients in the United States alone, chronic kidney disease (CKD) is an independent risk factor for cardiovascular (CV) disease and increased CV mortality [1]–[4]. Recently, CKD has been shown to be the predominant contributor to mortality among patients with type 2 diabetes mellitus (T2DM) [5], which still carries a high absolute risk of CV mortality despite more aggressive treatment of modifiable risk factors [1], [6]–[10]. This persistently elevated mortality risk may in part be due to unrecognized diabetic subpopulations at increased risk as well as unrecognized novel risk factors.

Prior studies examining mortality and CV outcomes in diabetics with CKD have focused on patients with microalbuminuria or with an estimated glomerular filtration rate (eGFR) less than 60 mL/min/1.73 m^2^. The role of early CV prevention for T2DM patients with mild GFR impairment (eGFR 60–90 mL/min/1.73 m^2^) in the absence of albuminuria remains unclear. Understudied, this group of diabetics without microalbuminuria does not meet current definitions of CKD and is not considered to be at increased CV risk [2]–[4], [11], [12]. However, mild GFR impairment may also be associated with modestly increased risk of both subclinical CV disease and adverse CV outcomes [5], [13], [14].

Furthermore, consensus guidelines for lipid management in T2DM patients with microalbuminuria or moderate to severe CKD focus on lowering low-density lipoprotein cholesterol (LDL-C) [11], [12], [15], which does not account for the full profile of lipid derangements seen in renal disease. Past studies have shown that CKD, even with microalbuminuria and eGFR>60 mL/min/1.73 m^2^ [16], is associated with elevated triglycerides (TG) and reduced high-density lipoprotein (HDL) but only minimal changes in LDL-C levels [17]–[20]. Dyslipidemia associated with renal impairment is also characterized by higher levels of triglyceride-rich lipoproteins (TGRL), which have recently garnered resurgent interest as causal biomarkers of CVD [21]–[23]. Measures of TGRLs include very-low-density lipoprotein (VLDL) cholesterol and triglycerides, apolipoprotein B (apoB), and apolipoprotein C-III (apoC-III), a protein that blocks lipoprotein lipase (LPL) and thus decreases VLDL metabolism and clearance. [17], [18], [23]–[30] In addition, lipoprotein(a) [Lp(a)], a lipoprotein consisting of an apo(B) moiety covalently linked to apolipoprotein(a) [apo(a)] and an independent genetic and causal risk factor for CVD [31]–[38], is significantly elevated in CKD patients compared to control populations [20], [38]–[41].

The purpose of this study was to probe whether T2DM patients with mild GFR impairment have atherogenic lipid profiles compared to their diabetic counterparts with normal renal function. We examined the association of mild GFR impairment, defined as eGFR between 60 and 90 mL/min/1.73 m^2^, with elevations in specific plasma lipid fractions in the Penn Diabetes Heart Study (PDHS), a cohort study of patients with T2DM but without overt CV disease or moderate to severe CKD. We hypothesized that mild renal impairment by eGFR criteria is already associated with atherogenic lipoprotein distribution characterized by elevated TG, VLDL-C, apoB, apoC-III, as well as increased circulating Lp(a).

## Materials and Methods

### Study Population

The Penn Diabetes Heart Study (PDHS), as previously described [42]–[46], is a single-center cross-sectional observational cohort of 2118 patients with T2DM, enrolled between 2001 and 2011. Participants were recruited from primary care and endocrinology clinics affiliated with the Hospital of the University of Pennsylvania (HUP). The University of Pennsylvania (Penn) Institutional Review Board approved the study protocol, and all subjects gave written informed consent. Inclusion criteria were: 1) a clinical diagnosis of T2DM (defined as fasting blood glucose >126 mg/dl, 2-hour post-prandial glucose >200 mg/dl, or use of oral hypoglycemic agents/insulin in a subject greater than age 40 years); 2) age of 35–75 years; and 3) a negative pregnancy test (if female and of child-bearing age). Exclusion criteria were: 1) history of clinical CV disease defined by myocardial infarction (MI), documented angiographic coronary artery disease, positive stress test, coronary or peripheral revascularization, stroke, or transient ischemic attack; 2) insulin use prior to age 35; 3) renal insufficiency defined at the time of recruitment as serum creatinine greater than 2.5 mg/dL; 4) active infection or malignancy; and 5) weight more than 300 pounds. For the current study, subjects were additionally excluded if their urinary albumin to creatinine ratio (ACR) was greater than 3000 mg/g or if their serum creatinine-based eGFR, calculated by the Chronic Kidney Disease Epidemiology Collaboration (CKD-EPI) equation [47], was determined to be below 60 mL/min/1.73 m^2^, leaving final sample size of 1852 subjects.

### Data Collection

Study subjects were evaluated at the Clinical and Translational Research Center (CTRC) at HUP after a 12-hour fast. They completed a questionnaire regarding past medical, social, and family history as well as use of medications. Height, weight, waist and hip circumference, and bilateral resting systolic and diastolic blood pressures were measured. Samples of whole blood and urine were collected, processed, and stored (at −80 C). Complete blood count, basic metabolic panel, hemoglobin A1c, and microalbuminuria assays were performed at the clinical laboratories of HUP. Plasma insulin levels were measured at the core laboratory of the Penn Diabetes Center by radioimmunoassay (Linco Research, St. Charles, MO). Total cholesterol (TC), TG, HDL-C, and VLDL-C were measured enzymatically, while LDL-C was measured directly, after ultracentrifugation (β-centrifugation technique) in a Centers for Disease Control (CDC) certified lipid laboratory [42], [43], [45]. Lp(a) and apolipoproteins were measured by immunoturbidimetric assay (Wako Chemicals, U.S.A. Inc., Richmond, VA) on a Hitachi 912 autoanalyzer (Roche Diagnostics, Basel, Switzerland). Laboratory test results were generated by personnel blinded to the clinical characteristics of the study participants.

Hypertension was defined as meeting one of the following criteria: 1) self-reported history of hypertension; 2) use of anti-hypertensive medications; 3) documented systolic blood pressure measurement greater than 140 mmHg; or 4) documented diastolic blood pressure measurement greater than 90 mmHg. Fasting homeostasis model assessment-estimated insulin resistance (HOMA-IR) was calculated using the following equation: (glucose [mg/dL] × insulin [µIU/mL]/405) [48]. eGFR was calculated based on the CKD-EPI equation [47]. Urinary ACR for each subject, reported in mg/g, was calculated by dividing spot urine albumin by spot urine creatinine concentrations.

### Statistical Analysis

For descriptive data and unadjusted analyses of lipid profiles, the study population was divided into two groups based on eGFR 60–90 vs. eGFR>90 ml/min/1.73 m^2^. Data distributions are reported as median and interquartile range (IQR) for continuous variables and as proportions for categorical variables. Unadjusted analyses compared data across the two different groups using the chi-squared test, Student's t-test, and the Wilcoxon rank sum test. Data for unadjusted analyses were also presented separately according to race, which has been shown to influence lipid profiles [49].

Lipid biomarkers with skewed distributions were log-transformed for multivariable analysis. Multivariable associations of lipid parameters with eGFR were analyzed separately as a continuous outcome and as a binary outcome for the groups 60 mL/min/1.73 m^2^ <eGFR <90 mL/min/1.73 m^2^ and eGFR>90 mL/min/1.73 m^2^. Associations were assessed in the following incremental logistic regression models: Model 1 was adjusted for age, race, and gender; Model 2 was also adjusted for body mass index (BMI), hypertension, and use of lipid-lowering medications; Model 3 was further adjusted for duration on insulin, hemoglobin A1c, and HOMA-IR; and Model 4 was additionally adjusted for urinary ACR. In addition, because Lp(a) values >30 mg/dL are associated with CV disease [36], logistic regression was performed on Lp(a) as a binary variable using 30 mg/dL as a cut-off value. Interactions between eGFR and race, gender, hypertension, and urinary ACR in association with Lp(a) were probed through interaction terms incorporated into the fully adjusted regression models. Correction for multiple testing was not performed given the clinical correlation among outcome variables and the complementary nature of the following multivariable models. Statistical analyses were performed using STATA version 13.0 software (Stata Corps, College Station, TX).

## Results

### Characteristics of Study Population by eGFR Profiles and Race

Baseline characteristics of this sample of PDHS subjects, divided according to eGFR groups of 60–90 and >90 mL/min/1.73 m^2^, are presented in Table 1. The majority of the 1852 participants were male (63.7%). The higher eGFR group had a larger proportion of black participants (39.8% vs. 27.5%, p<0.001), higher fasting glucose levels (121 vs. 112 mg/dL, p<0.001), and slightly lower urinary ACR values (4.9 vs. 5.3 mg/g, p = 0.04).

**Table 1:** Baseline Characteristics by eGFR Category.

	eGFR>90 mL/min/1.73 m^2^	eGFR 60–90 mL/min/1.73 m^2^	P Value
Number	998	854	
Age	56 (50–62)	63 (56–68)	<0.001
*Gender*			0.027
Men	61.4%	66.4%	
Women	38.6%	33.6%	
*Race*			<0.001
White	54.1%	67.1%	
Black	39.8%	27.5%	
Other	6.1%	5.4%	
Hypertension	73%	81%	<0.001
Systolic BP (mmHg)	130 (121–140)	131 (121–143)	0.12
Diastolic BP (mmHg)	77 (72–83)	75 (71–81)	0.001
Waist Circumference (in)	106 (97–117)	107 (97–116)	0.95
BMI (kg/m^2^)	32 (28–37)	32 (28–36)	0.20
Glucose (mg/dL)	121 (96–155)	112 (94–138)	<0.001
Hemoglobin A1c (%)	6.8 (6.2–8)	6.7 (6.1–7.4)	<0.001
HOMA-IR	4.68 (3.04–7.87)	4.29 (2.77–6.68)	0.10
Serum Creatinine (mg/dL)	0.8 (0.7–0.89)	1.0 (0.9–1.1)	<0.001
BUN (mg/dL)	13 (11–16)	17 (14–19)	<0.001
eGFR (CKD-EPI)	101.2 (95.48–109.45)	78.82 (72.09–84.69)	<0.001
Urinary ACR (mg/g)	4.9 (3–18.2)	5.3 (3–17.5)	0.04
*Anti-Hypertensive Medications*			
ACE Inhibitor or ARB	56.1%	65.1%	<0.001
Diuretic	24.6%	33.7%	<0.001
CCB	14.6%	20.5%	0.001
Beta Blocker	12.0%	16.1%	0.01
*Lipid-Lowering Medications*			
Statin	51.4%	59.4%	<0.001
Fibrate	4.3%	7.5%	0.003
Niacin	5.6%	5.7%	0.77

Categorical variables are shown as proportions; continuous variables as median values (IQR). Abbreviations: BP blood pressure, BMI body mass index, HOMA-IR homeostatic model assessment of insulin resistance, BUN blood urea nitrogen, eGFR estimated glomerular filtration rate, CKD-EPI Chronic Kidney Disease Epidemiology Collaboration, ACR albumin to creatinine ratio, CCB calcium channel blocker.

Lipid profiles by unadjusted analysis, reported as median values for each lipid parameter in Table 2, also differed between the eGFR groups. Because race may influence lipid profiles, unadjusted analyses for each lipid fraction are also presented by race. Compared to the higher eGFR group, the group with reduced eGFR had lower median LDL-C (93 vs. 98, p = 0.02), lower median apoA-II (33 vs. 34, p = 0.02), lower median apoB (79 vs. 82, p = 0.004), and higher median apoC-III (11.6 vs. 10.8, p = 0.04). Across all subjects, median Lp(a) did not differ between eGFR groups (20 vs. 20, p = 0.5), but median plasma Lp(a) levels were higher among black participants in the lower eGFR group compared to black participants in the higher eGFR group (49 vs. 42, p = 0.02).

**Table 2:** Lipid and Lipoprotein Measurements by eGFR Category.

	eGFR (mL/min/1.73 m^2^), All Races		P Value	eGFR (mL/min/1.73 m^2^), White Subjects		P Value	eGFR (mL/min/1.73 m^2^), Black Subjects		P Value
	>90	60–90		>90	60–90		>90	60–90	
TC*	173 (149–198)	169 (146–196)	0.07	169 (148–195)	167 (145–194)	0.25	176 (153–200)	170 (151–197)	0.22
HDL-C*	46 (37–55)	45 (38–55)	0.82	44 (36–52)	44 (37–52)	0.65	48 (41–58)	49 (40–61)	.39
LDL-C*	98 (79–120)	93 (77–115)	0.02	94.5 (78–116)	92 (76–113)	0.25	104 (82–125)	96 (82–116)	0.08
VLDL-C*	24 (17–35)	25 (17–37)	0.44	26 (19–38)	27 (18–39)	0.61	20 (14–29)	22 (15–29)	0.71
TG*	113 (80–170)	116 (85–170)	0.20	129 (94–194)	132 (92–187)	0.53	92 (70–127)	99 (74–128)	0.49
**ApoA-I^§^**	130 (116–147)	129 (116–144)	0.40	129 (114–146)	129 (116–142)	0.62	134 (120–149)	135 (118–155)	0.62
**ApoA-II^§^**	34 (31–38)	33 (30–37)	0.02	34 (30–37)	33 (30–37)	0.22	34 (31–39)	34 (30–38)	0.15
**ApoB^§^**	82 (70–96)	79 (68–92)	0.004	82 (72–96)	80 (69–92)	0.03	83 (70–96)	78 (68–91)	0.06
**ApoC-III^§^**	10.8 (7.5–15.4)	11.6 (8.3–15.6)	0.04	12.1 (9–16.2)	12.2 (9.2–16.2)	0.71	8.75 (6.1–12.6)	10.2 (6.8–14.2)	0.06
**ApoE^§^**	3.9 (3.2–4.7)	3.8 (3.2–4.5)	0.24	3.8 (3.1–4.5)	3.7 (3.1–4.4)	0.38	4.1 (3.4–4.9)	4 (3.4–4.8)	0.62
Lp(a)*	20 (8–51)	20 (7–50)	0.5	11 (6–28)	12 (6–31)	0.51	42 (20–76)	49 (25–89)	0.02
**FFA^§^**	0.61 (0.47–0.77)	0.62 (0.49–0.79)	0.41	0.63 (0.48–0.79)	0.63 (0.49–0.79)	0.74	0.59 (0.45–0.74)	0.62 (0.5–0.76)	0.18

Data represent bivariate associations between eGFR category and each denoted lipid parameter. eGFR categories were further stratified by white and black race. Data reported as median values (IQR). P values analyzed by Wilcoxon Rank Sum test. Abbreviations: TC total cholesterol, HDL-C high density lipoprotein cholesterol, LDL-C low density lipoprotein cholesterol, VLDL-C very low density lipoprotein cholesterol, TG trigylcerides, Lp(a) lipoprotein(a), apoA-I apolipoprotein A-I, apoA-II apolipoprotein A-II, apoB apolipoprotein B, apoC-III apolipoprotein C-III, apoE apolipoprotein E, FFA free fatty acids. *N = 1852,^ §^N = 1439

### Associations of Lipid Fractions with eGFR in Multivariable Models

Lp(a) analyses are discussed separately below. Linear regression with eGFR (all values >60 mL/min/1.73 m^2^) and log-transformed values of lipid fractions demonstrated that eGFR was significantly associated with levels of LDL-C and apoC-III after adjusting for demographic factors (Table 3). Although the association of eGFR with LDL-C lost significance after further adjustment, the associations of eGFR with apoC-III remained highly significant in the fully adjusted model that also accounts for urinary ACR (p = 0.001). The associations between eGFR and TG as well as eGFR and VLDL-C was significant after adjusting for hemoglobin A1c, HOMA-IR, and duration on insulin, in addition to demographic factors, BMI, hypertension, and use of lipid-lowering medications (Table 3) and remained significant in fully adjusted models (TG p = 0.01, VLDL-C p = 0.02). No statistically significant relationship between apoB and eGFR was seen.

**Table 3:** Multivariable Associations Between eGFR Values and Lipid Parameters.

Lipid Parameter	Model 1		Model 2		Model 3		Model 4	
	Beta Coefficient	P Value	Beta Coefficient	P Value	Beta Coefficient	P Value	Beta Coefficient	P Value
**TC**	0.029	0.28	0.010	0.70	−0.024	0.34	−0.020	0.42
**HDL-C**	0.012	0.64	−0.004	0.869	0.012	0.61	0.016	0.52
**LDL-C**	**0.065**	**0.016**	0.044	0.083	0.015	0.556	0.015	0.56
**VLDL-C**	−0.037	0.16	−0.034	0.206	**−0.062**	**0.02**	**−0.061**	**0.02**
**TG**	−0.036	0.17	−0.024	0.342	**−0.068**	**0.009**	**−0.066**	**0.01**
**ApoA-I**	0.026	0.36	0.016	0.569	0.039	0.18	0.047	0.11
**ApoA-II**	0.012	0.70	0.028	0.359	0.035	0.27	0.040	0.21
**ApoB**	0.053	0.09	0.039	0.182	−0.007	0.81	−0.006	0.85
**ApoC-III**	**−0.075**	**0.02**	**−0.070**	**0.02**	**−0.111**	**0.001**	**−0.109**	**0.001**
**ApoE**	−0.001	0.97	−0.008	0.801	−0.023	0.48	−0.014	0.67
**Lp(a)**	**−0.085**	**<0.001**	**−0.089**	**<0.001**	**−0.089**	**<0.001**	**−0.087**	**0.001**
**FFA**	0.031	0.30	0.027	0.361	0.024	0.44	0.022	0.47

Data represent standardized coefficients of change in log-transformed values of listed lipid fractions for every 10 ml/min/1.73 m^2^ higher eGFR. Linear regression was performed in incremental models with the following co-variates.

Model 1: Age, gender, race.

Model 2: Age, gender, race, BMI, hypertension, lipid-lowering medications (statin, fibrate, niacin).

Model 3: Age, gender, race, BMI, hypertension, lipid-lowering medications, hemoglobin A1c, HOMA-IR, duration on insulin.

Model 4: Age, gender, race, BMI, hypertension, lipid-lowering medications, hemoglobin A1c, HOMA-IR, duration on insulin, urinary ACR.

*Definition of hypertension includes the use of anti-hypertensive medications.

**Statistically significant results are presented in bold.

**Figure 1:**
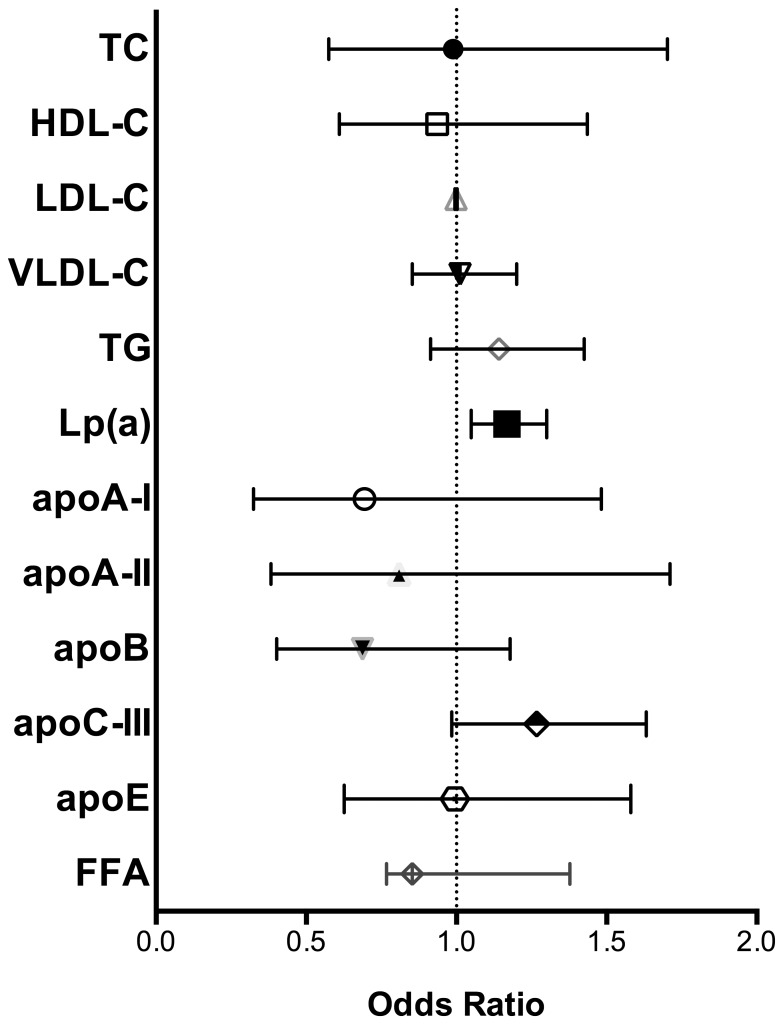
Odds of Lower eGFR Category by Lipid Parameters. Multivariable analysis adjusted for the following co-variates: age, race, gender, body mass index, hypertension (definition includes use of anti-hypertensive medications), use of lipid-lowering medication, hemoglobin A1c, HOMA-IR, duration on insulin, urinary albumin to creatinine ratio. Abbreviations: TC total cholesterol, HDL-C high density lipoprotein cholesterol, LDL-C low density lipoprotein cholesterol, VLDL-C very low density lipoprotein cholesterol, TG trigylcerides, Lp(a) lipoprotein(a), apoA-I apolipoprotein A-I, apoA-II apolipoprotein A-II, apoB apolipoprotein B, apoC-III apolipoprotein C-III, apoE apolipoprotein E, FFA free fatty acids.

When eGFR as a binary outcome (eGFR>90 vs. 60–90 mL/min/1.73 m^2^) underwent logistic regression with the log-transformed lipid fractions, associations between eGFR categories and apoB reached significance controlling for demographic factors, metabolic parameters, and use of lipid-lowering medications (Table 4). The association with apoB, however, weakened after further adjustment for glycemic measures and urinary ACR but maintained a trend towards significance (p = 0.17). The trends seen with VLDL-C, TG, and apoC-III when eGFR was treated as a continuous outcome were not observed when eGFR was analyzed by logistic regression using a binary outcome (eGFR>90 vs. 60–90 mL/min/1.73 m^2^) (Fig. 1).

**Table 4:** Multivariable Associations Between Lower eGFR Category and Lipid Parameters.

Lipid Parameter	Model 1		Model 2		Model 3		Model 4	
	OR (95% CI)	P Value	OR (95% CI)	P Value	OR (95% CI)	P Value	OR (95% CI)	P Value
**TC**	0.80 (0.50–1.29)	0.36	0.82 (0.49–1.36)	0.44	1.03 (0.60–1.77)	0.92	0.99 (0.58–1.70)	0.97
**HDL-C**	0.86 (0.58–1.28)	0.47	1.01 (0.67–1.52)	0.97	0.96 (0.63–1.48)	0.87	0.94 (0.61–1.44)	0.76
**LDL-C**	1.00 (0.99–1.00)	0.15	1.00 (0.99–1.00)	0.18	1.00 (0.99–1.00)	0.50	1.00 (0.99–1.00)	0.53
**VLDL-C**	1.01 (0.87–1.19)	0.87	0.98 (0.84–1.15)	0.83	1.02 (0.86–1.21)	0.84	1.14 (0.91–1.43)	0.24
**TG**	1.10 (0.90–1.34)	0.37	1.03 (0.84–1.23)	0.79	1.15 (0.92–1.44)	0.84	1.01 (0.85–1.20)	0.89
**ApoA-I**	0.79 (0.39–1.59)	0.50	0.88 (0.43–1.81)	0.73	0.76 (0.36–1.62)	0.48	0.69 (0.32–1.48)	0.35
**ApoA-II**	1.00 (0.50–2.00)0	0.99	0.88 (0.44–1.78)	0.73	0.85 (0.40–1.79)	0.67	0.81 (0.38–1.71)	0.58
**ApoB**	**0.58 (0.36–0.93)**	**0.02**	**0.55 (0.33–0.91)**	**0.021**	0.70 (0.41–1.19)	0.19	0.69 (0.40–1.18)	0.17
**ApoC-III**	1.15 (0.91–1.46)	0.24	1.13 (0.89–1.43)	0.33	1.28 (0.99–1.65)	0.06	1.27 (0.98–1.63)	0.07
**ApoE**	0.93 (0.61–1.42)	0.73	0.93 (0.60–1.44)	0.74	1.06 (0.67–1.68)	0.82	0.99 (–.63–1.58)	0.98
**Lp(a)**	**1.16 (1.05–1.28)**	**0.004**	**1.17 (1.06–1.30)**	**0.002**	**1.17 (1.05–1.31)**	**0.004**	**1.17 (1.04–1.30)**	**0.005**
**FFA**	1.00 (0.76–1.31)	0.98	0.99 (0.75–1.31)	0.94	1,02 (0.76–1.37)	0.88	1.03 (0.77–1.38)	0.85

Data represent odds of lower eGFR category (mild renal impairment) with log-transformed values of lipid fractions. Logistic regression was performed in incremental models with the following co-variates.

Model 1: Age, gender, race.

Model 2: Age, gender, race, BMI, hypertension, lipid-lowering medications (statin, fibrate, niacin).

Model 3: Age, gender, race, BMI, hypertension, lipid-lowering medications, hemoglobin A1c, HOMA-IR, duration on insulin.

Model 4: Age, gender, race, BMI, hypertension, lipid-lowering medications, hemoglobin A1c, HOMA-IR, duration on insulin, urinary ACR.

*Definition of hypertension includes the use of anti-hypertensive medications.

**Statistically significant results are presented in bold.

### Higher Lp(a) Levels Are Associated with Mild GFR Impairment

Across all incremental multivariable models, the lower eGFR group maintained a robust and consistent association with higher plasma Lp(a) levels (OR 1.17, 1.05–1.30, p = 0.005). Thus, the inverse association remained statistically significant even with adjustment for race, glycemic parameters, and urinary ACR (Table 4). Using a cut-point in circulating Lp(a) levels (at 30 mg/dL), values above which had previously been found to be associated with CV disease [36], logistic regression in incremental models again demonstrated a statistically significant and consistent association (e.g., in fully adjusted model OR 1.35, 1.06–1.71, p = 0.013) between mild GFR impairment (eGFR 60–90 mL/min/1.73 m^2^) and this clinical definition of elevated circulating Lp(a) levels (Table 5). Interaction terms between eGFR and race, gender, hypertension, and urinary ACR in association with Lp(a) were not statistically significant.

**Table 5:** Association Between Lower eGFR Category and Lp(a)>30 mg/dL.

Multivariable Model	Odds Ratio (95% CI)	P Value
Model 1	1.33 (1.06–1.67)	0.014
Model 2	1.34 (1.07–1.69)	0.012
Model 3	1.36 (1.07–1.72)	0.011
Model 4	1.35 (1.06–1.71)	0.013

Data represent odds of lower eGFR category with plasma Lp(a) greater than 30 mg/dL. Logistic regression performed in incremental models with the following co-variates.

Model 1: Age, gender, race.

Model 2: Age, gender, race, BMI, hypertension, lipid-lowering medications (statin, fibrate, niacin).

Model 3: Age, gender, race, BMI, hypertension, lipid-lowering medications, hemoglobin A1c, HOMA-IR, duration on insulin.

Model 4: Age, gender, race, BMI, hypertension, lipid-lowering medications, hemoglobin A1c, HOMA-IR, duration on insulin, urinary ACR.

*Definition of hypertension includes the use of anti-hypertensive medications.

## Discussion

In this study of diabetic individuals, we demonstrate that mild GFR impairment is indeed associated with an atherogenic lipid profile, primarily through its strong association with elevated plasma Lp(a) levels. Previous studies with smaller cohorts have shown mixed results in evaluating the correlation between plasma Lp(a) and markers of renal disease, including reduced eGFR and albuminuria [39], [50]. Utilizing a large, well-characterized cohort of individuals with T2DM, our study is among the first to demonstrate a robust association between higher plasma Lp(a) levels and mildly reduced eGFR independent of race, glycemic control, and albuminuria. This significant association was also observed when Lp(a) levels were dichotomized at 30 mg/dL, a clinically relevant cutoff in Lp(a). Thus, Lp(a) levels greater than 30 mg/dL are associated with increased risk of CV disease [36] and in our study are associated with the lower eGFR.

Among diabetic individuals with an eGFR greater than 60 mL/min/1.73 m^2^, the association between higher plasma Lp(a) and reduced eGFR may have potential implications in the care of T2DM patients with mild GFR impairment. Lp(a) levels are well established to be elevated in moderate to severe CKD patients, [20], [38]–[41] and here we show that they are also elevated in the setting of mild GFR impairment regardless of albuminuria status. Recent studies have identified Lp(a) as an independent genetic and causal risk factor for CV disease[31]–[38] with several studies showing that higher plasma levels of Lp(a) and genetic variation in the LPA gene that raise Lp(a) levels increase the risk of myocardial infarction (MI) in patients with and without established coronary heart disease [31], [37], [38], [51]. Thus, specific therapeutic targeting of Lp(a) is under development for treatment of heart disease. Our findings suggest that elevated Lp(a) levels might contribute even from an early stage of CKD to the higher incidence of CV disease in patients with CKD [52]. Specifically, the association of elevated plasma Lp(a) with eGFR in the 60–90 ml/min/1.73 m^2^ range independent of albuminuria status suggests that diabetic patients with even mild renal impairment may have more cardiovascular risk than previously thought. This also raises the possibility of targeting of Lp(a) as a preventative strategy for lowering CVD in CKD patients.

Our findings for Lp(a) contradict a previous study that found Lp(a) to be positively associated with albuminuria [39] and separate work that found an association between Lp(a) and albuminuria but not with eGFR in a smaller cohort primarily of non-diabetic patients [50]. In the PDHS cohort, the association between Lp(a) and eGFR in both linear and logistic regression models was consistent and robust to adjustment for urinary ACR. In contrast, the association of Lp(a) with urinary ACR in the PDHS sample did not reach statistical significance in any models (data not shown). Overall, our results indicate that mildly reduced eGFR alone in T2DM patients is associated with higher levels of a this pro-atherogenic lipid moiety and that the sub-population of diabetics with eGFR between 60 and 90 mL/min/1.73 m^2^ but without albuminuria may warrant further study of their CV disease risk.

Although our study demonstrates an inverse relationship between eGFR and Lp(a), the mechanism behind this association remains unknown. Prior Mendelian randomization studies established causality between CV disease and genetic variations in the *LPA* gene [31], [37]. However, the causal direction between plasma Lp(a) and renal impairment is unclear. One study showed that plasma Lp(a) levels, which were not lowered by hemodialysis, decreased rapidly in CKD patients after renal transplantation, suggesting a role for the kidney in Lp(a) catabolism [52]. Another human study demonstrated a renovascular arteriovenous difference of −9% in Lp(a) concentrations as well as the presence of apo(a) fragments in urine, supporting the kidney's involvement in Lp(a) 's clearance from circulation [53]. Thus, renal disease *per se* may lead to increases in circulating Lp(a) and account for our observed association of Lp(a) levels with mild GFR impairment. However, it remains unclear whether Lp(a) also plays a role in the development and progression of renal disease. A prior study of 49 non-diabetic individuals with moderate to severe CKD did not find a statistically significant association between plasma Lp(a) levels and progression of renal disease [54]. However, it utilized a small study sample with a majority of subjects having already moderate renal impairment due to chronic glomerulonephritis; thus these subjects may have had stronger competing risks for CKD progression. A larger Chronic Renal Insufficiency Cohort (CRIC) study also did not find a statistically significant association between Lp(a) levels and CKD progression [55] but again had participants with more severe renal disease than the PDHS subjects. Because these studies focused on populations with different characteristics from the PDHS cohort, they do not necessarily counter the possibility that Lp(a) may play a causal role in early CKD in diabetics, although determination of causality is beyond the scope of our study.

Unlike Lp(a), other lipid fractions we studied did not demonstrate a robust association with mild GFR impairment (eGFR <90 but >60 mL/min/1.73 m^2^). Although we had hypothesized that elevated plasma levels of TG, VLDL-C, apoB, and apoC-III would be associated with mild GFR impairment, the inverse relationship between eGFR and TG, VLDL, and apoC-III seen on linear regression did not remain statistically significant on logistic regression with mild GFR impairment as an outcome. This finding is consistent with greater power in modeling eGFR as a continuous outcome variable compared to examining eGFR outcomes as collapsed binary variables.

Prior studies have demonstrated that TGRLs and multiple apolipoprotein fractions were associated with CKD, but these studies generally examined populations with lower mean eGFR and higher urinary ACR values than the PDHS sample [20], [25], [26], [29], [30]. Also, their analyses were either not adjusted or did not adjust for hypertension, glycemic control, and insulin resistance. Here we show by linear regression analyses that TGRLs are associated with eGFR in the PDHS sample. This trend could be mediated by increased apoC-III as higher apoC-III levels in the setting of CKD is likely to impair LPL activity and decrease TG and VLDL metabolism. Currently, therapies targeting the apoC-III pathway are being developed [56], and further studies on apoC-III and outcomes in mild CKD are needed.

Our work has several strengths. This study, including a substantial proportion of women and minorities, is the largest with the unique focus on CV biomarkers in a population (mild GFR impairment) that may be under-recognized for increased CV risk. Other studies on Lp(a) in renal disease have primarily included patients on dialysis or with late-stage CKD who already have established increased CV risk and excess all-cause mortality. Our findings for Lp(a) in multivariable analyses were robust, with control for confounding variables of clinical relevance to CKD as well as CV disease. Our study also has some weaknesses. The cross-sectional nature of our study does not allow for the establishment of a causal direction in the associations we found between eGFR and the lipid parameters of interest. Other weaknesses include the use of eGFR rather than measured GFR or cystatin-C, recruitment of patients from a small geographical area, absence of a non-diabetic comparison group, absence of isoform and genotypic data for *LPA*.

In summary, our data show that in diabetic individuals mild renal impairment has a robust association with elevated Lp(a) levels and also appears to associate with increased apoC-III which ultimately leads to elevated atherogenic TGRLs. Both of these lipid mediators are causal in CV disease and also are promising therapeutic targets currently under development. Whether these lipid pathways represent specific targets for increased risk of CV disease in CKD population remains to be established. Future studies are warranted to assess potential benefit of early preventive interventions even in populations with mild CKD where these early lipid abnormalities may increase later risk of CV events. Finally, genomic studies are required to address whether *LPA* variants associated with CV disease are also associated with development and progression of CKD in order to establish whether Lp(a) is a causal risk factor for CKD and hence a potential future therapeutic target for reducing renal outcomes as well as CV event.

## Supporting Information

S1 Table **Correlation Matrix of All Lipid Parameters.** Spearman's correlation coefficient is reported for each lipid parameter with all other corresponding lipid parameters. Coefficients with p<0.05 are bolded. Abbreviations: TC total cholesterol, HDL-C high density lipoprotein cholesterol, LDL-C low density lipoprotein cholesterol, VLDL-C very low density lipoprotein cholesterol, TG trigylcerides, Lp(a) lipoprotein(a), apoA-I apolipoprotein A-I, apoA-II apolipoprotein A-II, apoB apolipoprotein B, apoC-III apolipoprotein C-III, apoE apolipoprotein E, FFA free fatty acids.
(DOCX)

S2 Table **Multivariable Associations Between eGFR Values and Lipid Parameters.** Data represent standardized coefficients of change in log-transformed values of listed lipid fractions for every 10 ml/min/1.73 m2 higher eGFR. Linear regression was performed in incremental models with the following co-variates: age, gender, race, BMI, hypertension, lipid-lowering medications, hemoglobin A1c, HOMA-IR, duration on insulin, urinary ACR, and all other lipid parameters found to have significant associations with eGFR (those listed in this table).
(DOCX)

S3 Table **De-identified data from this dataset were used for the analysis presented in this manuscript.** The second worksheet of the Excel file contains a key to the binary coding of variables.
(XLS)
