# Supplementary material for: Plasma Lipoprotein(a) Levels Are Associated with Mild Renal Impairment in Type 2 Diabetics Independent of Albuminuria
Source: PLoS One. 2014 Dec 9;9(12):e114397. doi: 10.1371/journal.pone.0114397 (PMC4260843; doi:10.1371/journal.pone.0114397)
Supplement: S1 Table — Correlation Matrix of All Lipid Parameters. Spearman's correlation coefficient is reported for each lipid parameter with all other corresponding lipid parameters. Coefficients with p<0.05 are bolded. Abbreviations: TC total cholesterol, HDL-C high density lipoprotein cholesterol, LDL-C low density lipoprotein cholesterol, VLDL-C very low density lipoprotein cholesterol, TG trigylcerides, Lp(a) lipoprotein(a), apoA-I apolipoprotein A-I, apoA-II apolipoprotein A-II, apoB apolipoprotein B, apoC-III apolipoprotein C-III, apoE apolipoprotein E, FFA free fatty acids. (DOCX) [file pone.0114397.s001.docx]

**Table S1. Correlation Matrix of All Lipid Parameters**

|  | **TC** | **HDL-C** | **LDL-C** | **VLDL-C** | **TG** | **ApoA-I** | **ApoA-II** | **ApoB** | **ApoC-III** | **ApoE** | **Lp(a)** | **FFA** |
| --- | --- | --- | --- | --- | --- | --- | --- | --- | --- | --- | --- | --- |
| **TC** | **1.00** |  |  |  |  |  |  |  |  |  |  |  |
| **HDL-C** | **0.22** | **1.00** |  |  |  |  |  |  |  |  |  |  |
| **LDL-C** | **0.86** | **0.06** | **1.00** |  |  |  |  |  |  |  |  |  |
| **VLDL-C** | **0.38** | **-0.41** | **0.13** | **1.00** |  |  |  |  |  |  |  |  |
| **TG** | **0.30** | **-0.48** | **0.10** | **0.88** | **1.00** |  |  |  |  |  |  |  |
| **ApoA-I** | **0.25** | **0.77** | 0.05 | **-0.16** | **-0.19** | **1.00** |  |  |  |  |  |  |
| **ApoA-II** | **0.24** | **0.41** | **0.08** | **0.08** | **0.05** | **0.57** | **1.00** |  |  |  |  |  |
| **ApoB** | **0.80** | **-0.15** | **0.82** | **0.46** | **0.42** | -0.04 | **0.07** | **1.00** |  |  |  |  |
| **ApoC-III** | **0.36** | **-0.06** | **0.16** | **0.50** | **0.58** | **-0.15** | 0.01 | **0.34** | **1.00** |  |  |  |
| **ApoE** | **0.45** | 0.04 | **0.25** | **0.45** | **0.38** | **0.22** | **0.24** | **0.39** | **0.25** | **1.00** |  |  |
| **Lp(a)** | **0.13** | **0.20** | **0.16** | **-0.16** | **-0.23** | **0.08** | -0.02 | 0.04 | **-0.12** | 0.03 | **1.00** |  |
| **FFA** | **0.17** | **0.09** | **0.07** | **0.15** | **0.18** | **0.15** | 0.03 | **0.13** | 0.15 | **0.18** | -0.03 | **1.00** |

Spearman’s correlation coefficient is reported for each lipid parameter with all other corresponding lipid parameters. Coefficients with p < 0.05 are bolded. Abbreviations: TC total cholesterol, HDL-C high density lipoprotein cholesterol, LDL-C low density lipoprotein cholesterol, VLDL-C very low density lipoprotein cholesterol, TG trigylcerides, Lp(a) lipoprotein(a), apoA-I apolipoprotein A-I, apoA-II apolipoprotein A-II, apoB apolipoprotein B, apoC-III apolipoprotein C-III, apoE apolipoprotein E, FFA free fatty acids.
