# Supplementary material for: Plasma Lipoprotein(a) Levels Are Associated with Mild Renal Impairment in Type 2 Diabetics Independent of Albuminuria
Source: PLoS One. 2014 Dec 9;9(12):e114397. doi: 10.1371/journal.pone.0114397 (PMC4260843; doi:10.1371/journal.pone.0114397)
Supplement: S2 Table — Multivariable Associations Between eGFR Values and Lipid Parameters. Data represent standardized coefficients of change in log-transformed values of listed lipid fractions for every 10 ml/min/1.73 m2 higher eGFR. Linear regression was performed in incremental models with the following co-variates: age, gender, race, BMI, hypertension, lipid-lowering medications, hemoglobin A1c, HOMA-IR, duration on insulin, urinary ACR, and all other lipid parameters found to have significant associations with eGFR (those listed in this table). (DOCX) [file pone.0114397.s002.docx]

**Table S2.** **Multivariable Associations Between eGFR Values and Lipid Parameters**

| **Lipid Parameter** | **Beta Coefficient** | **P-Value** |
| --- | --- | --- |
| **Lp(a)** | -0.107 | <0.001 |
| **ApoC-III** | -0.063 | 0.026 |
| **VLDL-C** | 0.004 | 0.827 |
| **TG** | -0.012 | 0.468 |

Data represent standardized coefficients of change in log-transformed values of listed lipid fractions for every 10 ml/min/1.73m^2^ higher eGFR. Linear regression was performed in incremental models with the following co-variates: age, gender, race, BMI, hypertension, lipid-lowering medications, hemoglobin A1c, HOMA-IR, duration on insulin, urinary ACR, and all other lipid parameters found to have significant associations with eGFR (those listed in this table).
